# *In silico* approaches for predicting the half-life of natural and modified peptides in blood

**DOI:** 10.1371/journal.pone.0196829

**Published:** 2018-06-01

**Authors:** Deepika Mathur, Sandeep Singh, Ayesha Mehta, Piyush Agrawal, Gajendra P. S. Raghava

**Affiliations:** 1 Bioinformatics Centre, CSIR-Institute of Microbial Technology, Chandigarh, India; 2 Computational Biology, Indraprastha Institute of Information Technology, New Delhi, India; University of Cambridge, UNITED KINGDOM

## Abstract

This paper describes a web server developed for designing therapeutic peptides with desired half-life in blood. In this study, we used 163 natural and 98 modified peptides whose half-life has been determined experimentally in mammalian blood, for developing in silico models. Firstly, models have been developed on 261 peptides containing natural and modified residues, using different chemical descriptors. The best model using 43 PaDEL descriptors got a maximum correlation of 0.692 between the predicted and the actual half-life peptides. Secondly, models were developed on 163 natural peptides using amino acid composition feature of peptides and achieved a maximum correlation of 0.643. Thirdly, models were developed on 163 natural peptides using chemical descriptors and attained a maximum correlation of 0.743 using 45 selected PaDEL descriptors. In order to assist researchers in the prediction and designing of half-life of peptides, the models developed have been integrated into PlifePred web server (http://webs.iiitd.edu.in//raghava/plifepred/).

## Introduction

The technological advances have led to the revival of interest of the pharmaceutical industry in peptide-based therapeutics [1]. Peptides show diverse therapeutic properties [2,3] like anticancer [4], antimicrobial [5,6], antiparasitic [7], cell penetrating [8,9], antihypertensive [10], tumor homing [11]. The peptides have a number of advantages over small molecule-based drugs that include high specificity and low side effects [1,12]. Despite many advantages, therapeutic peptides still face many roadblocks on the road to the pharmaceutical market. The major hurdle that is blocking the path of development of therapeutic peptides is their short half-life due to their susceptibility to enzymatic degradation that reduces their bioavailability. Different routes of peptide deliveries have been explored that include intranasal [13], transdermal [14], oral [15], pulmonary [16], rectal [17]. The parenteral route of peptide delivery is preferred over other routes of administration for efficient systemic delivery as it prevents cleavage of peptides by the gastrointestinal enzymes.

In the past, numerous attempts have been made to increase the half-life of peptides in blood that includes cyclization of peptides, incorporation of modified residues and terminal modifications [18]. These methods not only enhance the in-vivo half-life but also increase bioavailability. Determination of half-life of novel peptides in blood is one of the major challenges in understanding their stability. The experimental techniques to determine the half-life of peptides are well established and highly accurate. Unfortunately, these experimental techniques are costly, cumbersome and time-consuming. Therefore, alternate methods are required for estimating the half-life of peptides. An *in silico* method to predict and design half-life of peptides in blood will be an invaluable tool for the researchers working in the field of therapeutic peptides. Previously, computational tools have been developed for predicting the half-life of proteins. ProtLifePred [http://protein-n-end-rule.leadhoster.com/] and ProtParam [19] are based on the N-end rule and predict the half-life of proteins in E.coli, S. cerevisiae and mammalian cells. The stability of HIV-derived peptides in the cytosol of human peripheral blood mononuclear cells can be judged using the Stability Prediction tool [20]. SprotP server [21] identifies proteins with a half-life less than 30 minutes in human embryonic kidney 293T cells. Recently, our group developed a web server HLP [22] for predicting half-life of peptides in the intestine-like environment.

To the best of the authors’ knowledge no *in silico* method has been developed to predict the half-life of peptides in mammalian blood. Thus, we made a systematic attempt to understand the nature of peptides having long life and short life in mammalian blood. In the present study, we have developed *in silico* models using various machine learning techniques and features namely, amino acid composition, dipeptide composition, binary profile, atom composition and chemical descriptors to predict the half-life of peptides in blood.

## Methods

### Dataset

**Fig 1:**
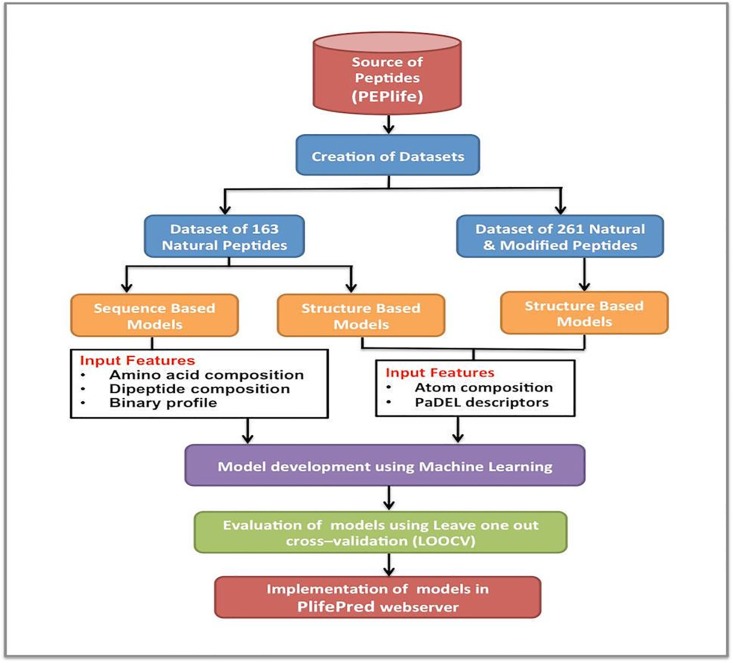
Workflow of PlifePred.

We extracted sequences and structures of the experimentally determined half-life of peptides from PEPlife [23], which is a database of the half-life of 2230 peptides in various environments like blood, urine, intestinal, kidney and brain homogenates, various cell lines and media like PBS, etc. We used following procedure to derive our dataset. Firstly, we extracted the peptides whose half-life had been experimentally validated in mammalian blood from PEPlife and obtained 1392 entries. Secondly, we removed all peptides having number of residues more than 50 or less than 5. Thirdly, we removed all those peptides having half-life more than 24 hours and less than 20 seconds. After the above filters, we got 1119 peptides having length from 5 to 50 and half-life from 20 seconds to 24 hours. Fourthly, from these 1119 peptides, we removed peptide sequences that had complex terminal modifications like PEGylation, biotinylation etc. or complex non-terminal modifications like sarcosine, β-alanine, etc. The peptides whose structures were not available in PDB database or PEPlife database were also removed; we got 682 peptides after this step. Finally, we got 261 unique peptides (See Supporting information pdb_files.zip) after removing redundancy, where no two peptides are identical. We called this dataset of 261 peptides as modified dataset as it contains natural and modified peptides. We also created dataset of natural peptides that contain only 163 natural peptides. The dataset consists of unique non-identical sequences, though few sequences may have up to 90% sequence similarity due to availability of limited dataset. Detailed information of the peptides containing natural as well as modified residues is given in Supporting Information file 261_natural+modified.xlsx whereas information of the peptides containing only natural residues is provided in Supporting Information file 163_natural.xlsx. Literature shows that even a single residue mutation or chemical modification in the peptide can change its half-life considerably [24,25]; so such peptides were retained in the dataset. In order to present half-life on a linear scale we have taken log2 of the half-life of peptides in seconds. The construction of datasets and the prediction approach followed is shown in Fig 1.

### Development of models

We used various machine learning techniques in this study for developing regression models. We implemented support vector machine (SVM) using SVM light software available at http://www.cs.cornell.edu/People/tj/svm_light/. SMOreg, Linear Regression, Gaussian Processes, IBk were implemented using Weka [26], a Java-based software package. In order to evaluate performance of models, we used leave-one-out cross-validation (LOOCV) technique. In LOOCV technique, for N number of peptides in the dataset, N-1 peptides are employed for training while the remaining one is used for testing. This process is repeated N times in order to test each peptide once. We also evaluated the performance of the model on 10% independent dataset of the natural peptides selected randomly. The experiment was perfromed 5 times and the average values were reported. In the present study, we used different types of features for developing models; the following is a brief description of the features.

- **Residue composition**: In this study, we used amino acid composition of peptides for developing models, where a vector of dimension 20 presents peptide. Similarly, models were also developed using dipeptide composition of peptides where the peptide is represented by a vector of dimension 400 [27,28].
- **Binary pattern**: The order and frequency of residues can be studied using the binary pattern profile of peptides [22]. To analyze the role of the terminal residues we took five residues from both the N and C terminus and calculated their binary profiles.
- **Atom composition**: It represents the frequency of 8 types of atoms (C, H, O, N, S, F, Cl, Br) present in the peptide sequence [29]. The atom composition was calculated from the SMILES of the peptide sequences, which includes the information of the chemical modifications as well as the amino acid.
- **Chemical descriptors**: Chemical descriptors are useful for developing QSAR models of peptides. We used PaDEL [30] which is an open source software for calculating more than 15,400 descriptors, consisting of 2D, 3D and fingerprints. To select the minimum number of descriptors that correlate to the half-life of peptides, we employed the CfsSubsetEval along with BestFirst modules of Weka.

## Results

### Analysis of peptides

**Fig 2:**
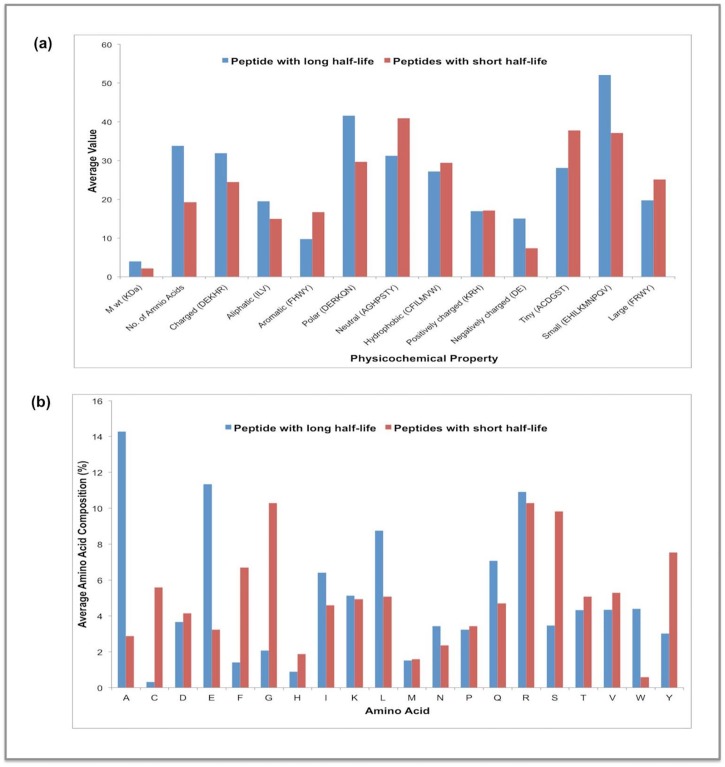
Comparison of the (a) physiochemical properties and (b) amino acid composition of top 20 peptides with the longest and shortest half-life.

We examined the physicochemical properties and amino acid composition of 20 peptides with the highest and the lowest half-lives (Fig 2). It was observed that peptides with long half-lives showed a high frequency of negatively charged (Glu) and small sized residues (Ala, Glu, Ile and Leu). These amino acids might be involved in stabilizing the half-life of peptides. The peptides with a short half-life are enriched in aromatic (Tyr and Phe) and neutral amino acids (Gly, His, Ser and Tyr). Previously, Morozumi et al., 2011 have shown that substitution of Glu with neutral amino acids resulted in lowering of the half-life of analogs of motilin-grehlin chimeric peptides [27]. We studied the distribution of half-life of the peptides with different sequence similarity present in our natural peptide dataset (S1 Fig) and observed that even the substitution of a single or double residue results in changing the half-life of the peptide significantly (S1 Table). The correlations between the half-lives of all 163 natural peptides with amino acid composition and physicochemical properties also show similar patterns (S2 and S3 Tables). It was observed that composition of amino acid Ala (a hydrophobic, non-polar residue) shows highest correlation followed by Glu (a negative charge residue) (S2 Table). In contrast, the composition of Phe (an aromatic residue) shows highest negative correlation with half-life of peptides. Previous studies have also reported lowering of half-life in peptides enriched in aromatic amino acids[31–33].

### Prediction of half-life on the natural dataset

*In silico* models have been developed on 163 natural peptides, to predict the half-life of peptides using different types of sequence-based features (Table 1). The amino acid composition based regression model achieved a maximum Pearson’s correlation coefficient (R) of 0.643 with mean absolute error (MAE) 1.531. The dipeptide composition based model attained R of 0.640 with MAE of 1.539. The atom composition achieved R of 0.532. To analyze the role of the amino acids present at the termini of the sequence, the first five residues of the N-terminus and the 5 residues from the C-terminus were used to develop models. Amino acid composition of N5 reached R of 0.251 with MAE of 2.723 while C5 achieved R of 0.245 with MAE of 2.317. Dipeptide composition of N5 reached R of 0.163 while R of C5 was 0.230. The binary composition of N5 showed R = 0.174 with MAE of 2.515 while C5 reached R = 0.271, MAE being 2.304. To develop structure-based regression models we used 45 selected PaDEL descriptors (S4 Table) and applied various machine-learning techniques. The maximum R of 0.743 with MAE = 1.369 was achieved on SMOreg (Table 2). Performance of the model trained on PaDEL descriptors was also evaluated on the 10% independent dataset. We achieved R of 0.2 with MAE 1.646 and RMSE of 2.11. Detailed description of the features can be obtained from http://www.yapcwsoft.com/dd/padeldescriptor/.

**Table 1:** Performance of SVM based regression models on various input features on 163 natural peptide dataset.

Features	Residues in peptide	R	MAE	RMSE
**Amino acid composition**	All residues	0.643	1.531	2.186
	5 N-terminal	0.251	2.723	3.359
	5 C-terminal	0.245	2.317	2.825
**Dipeptide composition**	All residues	0.640	1.539	2.196
	5 N-terminal	0.163	2.767	3.299
	5 C-terminal	0.230	2.378	2.821
**Binary pattern**	5 N-terminal	0.174	2.515	2.958
	5 C-terminal	0.271	2.304	2.786
**Atom composition**	All residues	0.532	1.761	2.426

**Table 2:** Results of the performance of various machine-learning techniques using 45 selected PaDEL descriptors as input feature on 163 natural peptide dataset.

Methods	R	MAE	RMSE
**SVM**	0.734	1.503	1.992
**SMOreg**	0.743	1.369	1.932
**Linear Regression**	0.696	1.659	2.119
**Gaussian Processes**	0.561	1.804	2.389
**IBk**	0.515	1.913	2.789

### Prediction of half-life on the modified dataset

On the dataset with 261 sequences containing both modified and natural sequences, we used atom composition and PaDEL descriptors as input features. Atom composition attained R of 0.586 with MAE of 1.756. The 43 selected PaDEL features (S5 Table) achieved a maximum Pearson’s correlation coefficient of 0.692 with MAE = 1.564. The performances of the models of other machine learning techniques are given in Table 3.

**Table 3:** Results of the performance of various machine-learning techniques using 43 selected PaDEL descriptors as input feature on 261 peptides containing both natural and modified residues.

Methods	R	MAE	RMSE
**SVM**	0.692	1.564	2.075
**SMOreg**	0.618	1.671	2.254
**Linear Regression**	0.630	1.656	2.208
**Gaussian Processes**	0.575	1.750	2.292
**IBk**	0.471	1.949	2.751

### Implementation and description of web-server

In order to contribute to the community, we have implemented the models developed in the form of a freely accessible web server called ‘PlifePred’. Researchers can utilize this platform to predict and design the half-life of peptides. This web-server has two main modules-Natural and Modified. The Natural module has two sub-modules-Sequence Based and Structure Based. The Sequence Based module has three modules: Analog Generation, Batch Submission and Protein Scan. These modules will facilitate users with peptide composed of all natural residues in the sequence. Analog Generation module allows users to predict the half-life of a natural peptide and generates all possible single point mutation analogs along with the physiochemical properties, facilitating the scientific community in designing peptides with desired half-life and physiochemical properties. Batch Submission module assists users to screen peptide sequences in bulk and predicts half-life as well as physiochemical properties of the sequences. The Protein Scan tool allows users to submit a protein sequence and it predicts the half-life of overlapping peptides of a length chosen by the user along with their physiochemical properties, besides allowing generation of mutant peptides of peptide fragment selected by the user. The Structure Based module has two modules: Draw and File. In the Draw module, Marvin Draw applet has been integrated to facilitate users to draw and submit queries of desired peptide structures. Users with pdb files of their query peptide can use the File module to predict the half-life of peptides in blood. The Modified module also has Draw and File sub-modules which will be useful in the rational designing of the half-life of peptides with chemical modifications and non-natural amino acids. This module will be useful for users to study the effect on the half-life of peptides when different modifications are introduced within its sequence. The PlifePred web-server was implemented using HTML, PHP and Perl languages and is available at URL http://webs.iiitd.edu.in/raghava/plifepred/.

## Discussion

Despite the advantages of peptides over small drugs, many of them fail to reach the market because of their low stability in vivo as a result of degradation by proteases. The half-life of therapeutic peptides governs their bioavailability, biodistribution and their dosing regimen. In the wet-lab, it is a costly and time-consuming process to synthesize peptides and examine the effect of mutating different residues and the role of different chemical modifications on the desired peptide. To help researchers and expedite their research, in the present study, we have developed *in silico* models to predict the half-life of modified as well as natural peptide sequences. The models have been developed on the largest available dataset of experimentally validated half-life of peptides in blood. The structure-based models using chemical descriptors as input features gave the best results for both modified and natural dataset followed closely by the amino acid composition-based model on the natural dataset. We also benchmarked our result with the tools (ProtLifePred, ProtParam and HLP) already available in the literature and observed that none of them were able to outperformed our method. ProtLifePred and ProtParam showed R of 0.051 with MAE 35298 whereas HLP showed the R of 0.08 with MAE of 2821. One possible reason could be that these softwares are not specifically designed for predicting half-life of peptides present in blood. The compositional analysis revealed that the charge and size of peptides are important parameters governing peptide stability. The shorter half-life is observed in peptides rich in large and aromatic amino acids whereas peptides with negatively charged and small amino acids have a longer half-life. These results are concordant with the study performed by Sharma et al. for analyzing the half-life of peptides in intestine-like environment [22]. The models obtained in the present study have been integrated in a freely available web server ‘PlifePred’ to aid the scientific community in the rational designing of peptide half-life. PlifePred will be a useful resource to predict and study the effects of various mutations and modifications on the half-life of peptides in blood.

## Supporting information

S1 Table Showing half-life of peptides having high similarity; having one or two mutations. (PDF)

S2 Table Correlation between half-life of natural peptide dataset and amino acid composition. (PDF)

S3 Table Correlation between half-life of natural peptide dataset and physicochemical properties. (PDF)

S4 Table Descriptors used for the development of structure based models on natural dataset. (PDF)

S5 Table Descriptors used for the development of structure based models on modified dataset. (PDF)

S1 Fig Shows variation in half-life of peptides by box-plot for different clusters having sequence similarity in different range. (PDF)

S1 File 261_natural+modified.xlsx: Detailed information of the peptides containing natural as well as modified residues. (XLSX)

S2 File 163_natural.xlsx: Detailed information of the peptides containing only natural residues. (XLSX)

S3 File pdb_files.zip: Structures of the peptides used in the study. (ZIP)
