# Supplementary material for: In silico approaches for predicting the half-life of natural and modified peptides in blood
Source: PLoS One. 2018 Jun 1;13(6):e0196829. doi: 10.1371/journal.pone.0196829 (PMC5983457; doi:10.1371/journal.pone.0196829)
Supplement: S1 Table — (PDF) [file pone.0196829.s001.pdf]

**S1 Table: Showing half-life of peptides having high similarity; having one or two mutations.**

| Example | Peptide Sequences | Half-life in seconds | Number of mutations |
|---|---|---|---|
| **1** | FVPIFTYGELQRVQQRKESKKPPAKLAALKA | 596 | 1 |
| | FVPIFTYGELQRVQQRKESKKPPAELAALKA | 693 | |
| **2** | HSQGTFTSDYSKYLDSRRAQDFVQWLMNTKRNRNNIA | 36000 | 1 |
| | HSQGTFTSDYSKYLDSRRAQDFVQWLMNTKRNKNNIA | 720 | |
| **3** | TTWEAWDRAIAEYAARIEALIRASQEQQEKNEAELREL | 4320 | 2 |
| | TTWEAWDRAIAEYAARIEALIRAAQEQQEKNEAALREL | 58680 | |
