# Supplementary material for: In silico approaches for predicting the half-life of natural and modified peptides in blood
Source: PLoS One. 2018 Jun 1;13(6):e0196829. doi: 10.1371/journal.pone.0196829 (PMC5983457; doi:10.1371/journal.pone.0196829)
Supplement: S2 Table — (PDF) [file pone.0196829.s002.pdf]

S2 Table: Correlation between half-life of natural peptide dataset and amino acid composition.

Amino Acid	One Letter Code	Correlation with half-life
**Alanine**	A	0.41
**Glutamic acid**	E	0.34
**Tryptophan**	W	0.27
**Isoleucine**	I	0.20
**Leucine**	L	0.07
**Threonine**	T	0.04
**Asparagine**	N	0.01
**Aspartic Acid**	D	-0.01
**Methionine**	M	-0.02
**Arginine**	R	-0.02
**Histidine**	H	-0.03
**Glutamine**	Q	-0.05
**Cysteine**	C	-0.05
**Tyrosine**	Y	-0.08
**Serine**	S	-0.09
**Valine**	V	-0.09
**Proline**	P	-0.16
**Lysine**	K	-0.16
**Glycine**	G	-0.17
**Phenylalanine**	F	-0.23
