# Supplementary material for: In silico approaches for predicting the half-life of natural and modified peptides in blood
Source: PLoS One. 2018 Jun 1;13(6):e0196829. doi: 10.1371/journal.pone.0196829 (PMC5983457; doi:10.1371/journal.pone.0196829)
Supplement: S3 Table — (PDF) [file pone.0196829.s003.pdf]

S3 Table: Correlation between half-life of natural peptide dataset and physicochemical properties.

Physicochemical Property	Correlation with half-life
Number of Amino Acids	0.29
Molecular weight (Da)	0.29
Negatively charged (DE)	0.28
Aliphatic (ILV)	0.07
Tiny (ACDGST)	0.05
Charged (DEKHR)	0.05
Polar (DERKQN)	0.03
Hydrophobic (CFILMVW)	0.01
Small (EHILKMNPQV)	-0.01
Neutral (AGHPSTY)	-0.03
Large (FRWY)	-0.07
Aromatic (FHWY)	-0.09
Positively charged (KRH)	-0.14
