# Supplementary material for: In silico approaches for predicting the half-life of natural and modified peptides in blood
Source: PLoS One. 2018 Jun 1;13(6):e0196829. doi: 10.1371/journal.pone.0196829 (PMC5983457; doi:10.1371/journal.pone.0196829)
Supplement: S4 Table — (PDF) [file pone.0196829.s004.pdf]

S4 Table: Descriptors used for the development of structure based models on natural dataset.

S. No.	Descriptors	Class	Description
1	PubchemFP149	Pubchem Fingerprint	>= 1 unsaturated non-aromatic heteroatom-containing ring size 5
2	PubchemFP36	Pubchem Fingerprint	>= 8 S
3	PubchemFP375	Pubchem Fingerprint	C(~N)(~N)
4	PubchemFP700	Pubchem Fingerprint	O-C-C-C-C-C-O-C
5	EStateFP7	Estate fingerprint	[OD2H0](-*)-*
6	ExtFP228	Extended Fingerprint	Extends the Fingerprinter with additional bits describing ring features
7	ExtFP236	Extended Fingerprint	Extends the Fingerprinter with additional bits describing ring features
8	ExtFP816	Extended Fingerprint	Extends the Fingerprinter with additional bits describing ring features
9	ExtFP875	Extended Fingerprint	Extends the Fingerprinter with additional bits describing ring features
10	ExtFP992	Extended Fingerprint	Extends the Fingerprinter with additional bits describing ring features
11	FP1021	CDK fingerprint	Fingerprint of length 1024 and search depth of 8
12	FP12	CDK fingerprint	Fingerprint of length 1024 and search depth of 8
13	FP276	CDK fingerprint	Fingerprint of length 1024 and search depth of 8
14	FP286	CDK fingerprint	Fingerprint of length 1024 and search depth of 8
15	FP648	CDK fingerprint	Fingerprint of length 1024 and search depth of 8
16	FP702	CDK fingerprint	Fingerprint of length 1024 and search depth of 8
17	FP704	CDK fingerprint	Fingerprint of length 1024 and search depth of 8
18	FP773	CDK fingerprint	Fingerprint of length 1024 and search depth of 8
19	FP825	CDK fingerprint	Fingerprint of length 1024 and search depth of 8
20	FP924	CDK fingerprint	Fingerprint of length 1024 and search depth of 8
21	FPSA-3	Charged Partial Surface Area (CPSA)	PPSA-3 / total molecular surface area (3D)
22	GraphFP167	CDK graph only fingerprint	Specialized version of the Fingerprinter which does not take bond orders into account
23	GraphFP359	CDK graph only fingerprint	Specialized version of the Fingerprinter which does not take bond orders into account
24	GraphFP752	CDK graph only fingerprint	Specialized version of the Fingerprinter which does not take bond orders into account
25	GraphFP888	CDK graph only fingerprint	Specialized version of the Fingerprinter which does not take bond orders into account
26	GraphFP919	CDK graph only fingerprint	Specialized version of the Fingerprinter which does not take bond orders into account
27	KRFP2410	Klekota-Roth fingerprint	[!#1]N1[CH2][CH2][CH2][CH]1C(=O)[OH]

**28**	KRFP3767	Klekota-Roth fingerprint	CCN(CCO)C=O
**29**	KRFP480	Klekota-Roth fingerprint	[!#1][CH2][NH]C(=O)[!#1]
**30**	KRFP669	Klekota-Roth fingerprint	[!#1][CH3]
**31**	KRFP681	Klekota-Roth fingerprint	[!#1][NH][CH]([!#1])[CH2][CH2][CH3]
**32**	KRFP691	Klekota-Roth fingerprint	[!#1][NH][CH]([CH2][CH]([CH3])[CH3])C(=O)[OH]
**33**	KRFP7	Klekota-Roth fingerprint	[!#1][CH]([!#1])[CH2][CH2][CH3]
**34**	KRFPC3054	Klekota-Roth fingerprint count	Count of chemical substructures
**35**	MACCSFP104	MACCS fingerprint	('[!#6;!#1;!H0]~*~[CH2]~*',0), # QHACH2A
**36**	MACCSFP109	MACCS fingerprint	('*~[CH2]~[#8]',0), # ACH2O
**37**	MACCSFP160	MACCS fingerprint	('[C;H3,H4]',0), #CH3
**38**	MACCSFP66	MACCS fingerprint	('[#6]~[#6](~[#6])(~[#6])~*',0), # CC(C)(C)A
**39**	MACCSFP90	MACCS fingerprint	('[$([!#6;!#1;!H0]~*~*~[CH2]~*),$([!#6;!#1;!H0;R]1@[R]@[R]@[CH2;R]1),$([!#6;!#1;!H0]~[R]1@[R]@[CH2;R]1)]',0), # QHAACH2A
**40**	maxdssC	Maximum atom-type E-State: =C<	Maximum atom-type E-State: =C< (2D)
**41**	maxssCH2	Maximum atom-type E-State: -CH2-	Maximum atom-type E-State: -CH2- (2D)
**42**	RNCS	Charged partial surface area	Charged partial surface area (2D)
**43**	VC-5	Chi cluster	Chi cluster(2D) / Valence cluster, order 5
**44**	Weta3.eneg	WHIMDescriptor	Directional WHIM descriptor weighted by Mulliken atomic electronegativites
**45**	Wnu1.eneg	WHIMDescriptor	Directional WHIM descriptor weighted by Mulliken atomic electronegativites
