# Supplementary material for: In silico approaches for predicting the half-life of natural and modified peptides in blood
Source: PLoS One. 2018 Jun 1;13(6):e0196829. doi: 10.1371/journal.pone.0196829 (PMC5983457; doi:10.1371/journal.pone.0196829)
Supplement: S5 Table — (PDF) [file pone.0196829.s005.pdf]

S5 Table: Descriptors used for the development of structure based models on modified dataset.

S. No.	Descriptors	Class	Description
1	ExtFP28	Extended Fingerprint	Extends the Fingerprinter with additional bits describing ring features
2	ExtFP816	Extended Fingerprint	Extends the Fingerprinter with additional bits describing ring features
3	FP286	CDK fingerprint	Fingerprint of length 1024 and search depth of 8
4	FP307	CDK fingerprint	Fingerprint of length 1024 and search depth of 8
5	FP841	CDK fingerprint	Fingerprint of length 1024 and search depth of 8
6	FPSA-3	Charged Partial Surface Area (CPSA)	PPSA-3 / total molecular surface area (3D)
7	GraphFP48	CDK graph only fingerprint	Specialized version of the Fingerprinter which does not take bond orders into account
8	GraphFP546	CDK graph only fingerprint	Specialized version of the Fingerprinter which does not take bond orders into account
9	GraphFP752	CDK graph only fingerprint	Specialized version of the Fingerprinter which does not take bond orders into account
10	GraphFP839	CDK graph only fingerprint	Specialized version of the Fingerprinter which does not take bond orders into account
11	KRFP2264	Klekota-Roth fingerprint	[!#1]N([!#1])[CH3]
12	KRFP309	Klekota-Roth fingerprint	[!#1][CH2][CH]([CH3])[NH]C(=O)[!#1]
13	KRFP3375	Klekota-Roth fingerprint	CC(C)C=O
14	KRFP3668	Klekota-Roth fingerprint	CCC=C(C)C
15	KRFP382	Klekota-Roth fingerprint	[!#1][CH2][CH2][CH3]
16	KRFP4664	Klekota-Roth fingerprint	OC(=O)CCc1c[nH]c2ccccc12
17	KRFP608	Klekota-Roth fingerprint	[!#1][CH2]N([!#1])[CH3]
18	KRFP630	Klekota-Roth fingerprint	[!#1][CH2]N([CH3])C(=O)[!#1]
19	KRFP758	Klekota-Roth fingerprint	[!#1][NH][CH2][CH]([!#1])[!#1]
20	KRFPC3054	Klekota-Roth fingerprint count	Count of chemical substructures
21	KRFPC309	Klekota-Roth fingerprint count	Count of chemical substructures
22	KRFPC3375	Klekota-Roth fingerprint count	Count of chemical substructures
23	KRFPC466	Klekota-Roth fingerprint count	Count of chemical substructures
24	KRFPC4664	Klekota-Roth fingerprint count	Count of chemical substructures
25	KRFPC608	Klekota-Roth fingerprint count	Count of chemical substructures

**26**	KRFPC630	Klekota-Roth fingerprint count	Count of chemical substructures
**27**	KRFPC822	Klekota-Roth fingerprint count	Count of chemical substructures
**28**	MACCSFP104	MACCS fingerprint	('[!#6;!#1;!H0]~*~[CH2]~*',0), # QHACH2A
**29**	MACCSFP109	MACCS fingerprint	('*~[CH2]~[#8]',0), # ACH2O
**30**	MACCSFP68	MACCS fingerprint	('[!#6;!#1;!H0]~[!#6;!#1;!H0]',0), # QHQH
**31**	MACCSFP90	MACCS fingerprint	('[$([!#6;!#1;!H0]~*~*~[CH2]~*),$([!#6;!#1;!H0;R]1@[R]@[R]@[CH2;R]1),$([!#6;!#1;!H0]~[R]1@[R]@[CH2;R]1)]',0), # QHAACH2A
**32**	maxssNH	Atom-type E-state (Electrotopological state)	Maximum atom-type E-State: -NH- (2D)
**33**	minHdsCH	Atom-type E-state (Electrotopological state)	Minimum atom-type H E-State: =CH- (2D)
**34**	PubchemFP151	Pubchem Fingerprint	>= 2 saturated or aromatic carbon-only ring size 5
**35**	PubchemFP172	Pubchem Fingerprint	>= 5 saturated or aromatic carbon-only ring size 5
**36**	PubchemFP193	Pubchem Fingerprint	>= 3 saturated or aromatic carbon-only ring size 6
**37**	PubchemFP194	Pubchem Fingerprint	>= 3 saturated or aromatic nitrogen-containing ring size 6
**38**	PubchemFP36	Pubchem Fingerprint	>= 2 saturated or aromatic nitrogen-containing ring size 8
**39**	PubchemFP690	Pubchem Fingerprint	O-C-C-C-C-C-O
**40**	PubchemFP700	Pubchem Fingerprint	O-C-C-C-C-C-O-C
**41**	Weta3.eneg	WHIMDescriptor	Directional WHIM descriptor weighted by Mulliken atomic electronegativites
**42**	WK.volume	WHIMDescriptor	Non-directional WHIM weighted by van der Waals volumes
**43**	Wnu1.eneg	WHIMDescriptor	Directional WHIM descriptor weighted by Mulliken atomic electronegativites
