# Supplementary material for: In silico approaches for predicting the half-life of natural and modified peptides in blood
Source: PLoS One. 2018 Jun 1;13(6):e0196829. doi: 10.1371/journal.pone.0196829 (PMC5983457; doi:10.1371/journal.pone.0196829)

Fig S1. Shows variation in half-life of peptides by box-plot for different clusters having sequence similarity in different range.

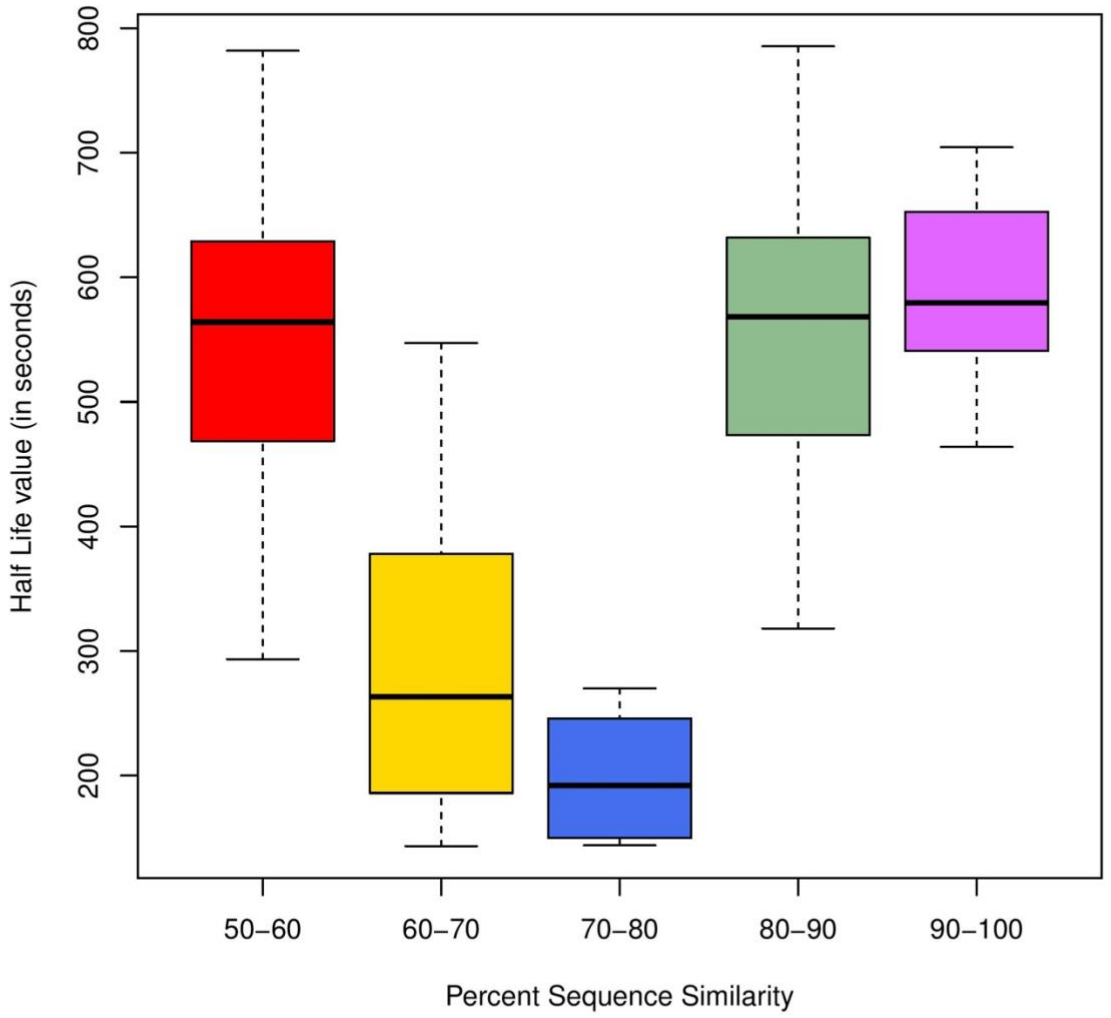

Supplement: S1 Fig — (PDF) [file pone.0196829.s006.pdf]
